# Meeting of the “American” Medical Association

**Published:** 1863-06

**Authors:** 


### MEETING OF THE "AMERICAN" MEDICAL ASSOCIATION.

Agreeably to the notice issued by the Chairman of the Committee of Arrangements, several of the States were represented in a convention in this city Tuesday, June 3d inst.

The convention was called to order at 11 o'clock by Wilson Jewell, of Pennsylvania, the First Vice President of the Association during the last three years. Officers occupied seats on the platform, with the exception of the President, who is deceased, and those from the seceding States. The following are the gentlemen who have held office since the year 1860:

*President*—Eli Ives, Connecticut.

*Vice Presidents*—Wilson Jewell, Pennsylvania; A. B. Palmer, Michigan; R. D. Arnold, Georgia; Joseph N. McDowell, Missouri.

*Secretaries*—S. G. Hubbard, Connecticut; H. A. Johnson, Illinois.

*Treasurer*—Caspar Wister, Pennsylvania.

Rev. R. L. Collier, pastor of the Wabash Avenue Methodist Episcopal Church, then invoked the Divine blessing upon the deliberations of the association.

The chairman of the Committee of Arrangements then welcomed the delegates in attendance, and read a report for the committee, giving their reason for its postponement the two previous years, and his for its assemblage at the present time. The report was adopted. The following delegates reported:

*Vermont*—G. N. Stiles, Lewis Emmons.

*Massachusetts*—Henry Cutter, Appleton Thorne, Edward Barton, James P. Lynde, Ebenezer Stone, P. J. Kendall, Benj. E. Cotting, Joshua Homans, John C. Dalton, M. D. Southwick, E. P. Abbe, John Green.

*New York*—Henry G. Davis, Guido Furman, Alden March, Daniel P. Bissel, James S. Whaley, Thomas C. Brinsmade, J. S. Sprague, C. C. F. Fay, Edward Hook, E. S. F. Arnold, E. W. Cherry, W. N. Blakeman, Howard Townsend, H. Nicholl, E. Tobie, H. S. Downs, C. C. Wyckoff, Alf. Underhill, J. H. Griscom, L. B. Cotes, Julius Homberger, C. H. Harvey, James McNaughton, Danl. Holmes.

*Connecticut*—Stephen G. Hubbard, L. N. Beardsley, B. H. Catlin, A. W. Barrows.

*New Jersey*—Wm. Pierson, Jr., D. M. Sayre, John Blain, Isaac S. Cramer.

*Delaware*—H. F. Askew, James Cooper.

*Pennsylvania*—Wilson Jewell, Wm. Mayberry, Edward Wallace, B. Richardson, John R. Thomas, E. H. Mason, Wm. L. Richardson, T. N. Trotz.

*Virginia*—J. C. Hupp.

*Ohio*—W. S. Battles, J. M. Taggert, W. W. Jones, A. H. Agard, K. G. Thomas, S. O. Almy, L. N. Lawson, W. B. Davis.

*Indiana*—B. S. Woodworth, A. M. Vickery, A. J. Erwin, A. P. Ferris, L. D. Personett, James Ferris, S. A. Freeman, L. D. Glazebrook, James F. Hibbard.

*Michigan*—A. B. Palmer, E. A. Egerry, H. O. Hitchcock, S. D. Richardson, Lewis Davenport.

*Illinois*—E. L. Holmes, Geo. K. Amerman, Edward Andrews, John Ten Broek, C. R. Parks, T. D. Fisher, Geo. W. Hall, David Prince, E. Andrews, N. Wright, W. O. Chamberlain, J. P. Ross, E. A. Steele, A. Fisher, M. J. Johnson, J. H. Hollister, D. Pierson, M. F. Dewitt, S. Wickersham, J. D. Rose, Henry Wing, Chas. Gorham, S. W. Noble, L. A. Hatch, T. F. Worrell, T. P. Haller, H. A. Johnson, R Spitler, G. Paoli, H. Noble, D. M. Tarker, Orrin Smith, A. J. Craine, T. K. Edmiston, J. J. Lulee.

*Wisconsin*—Chas. L. Stoddert, H. Adams, Hermon Van Dusen, E. S. Carr, Geo. D. Wilber.

*Iowa*—J. W. H. Baker, Samuel C. Lay, Jos. Sprague, D. L. McGugin.

*Kansas*—D. W. Stormont, C. A. Logan.

*Tennessee*—W. K. Boring.

*Army and Navy*—C. C. Cox, G. Simpson, A. R. Terry, John B. Porter, Benj. Palmer, M. K. Taylor, Ralph Isham.

The secretary then stated that the delegates could procure free return tickets by connecting themselves with the great canal convention, in session in the city, as delegates.

The delegates from the several States then resolved themselves into sub-committees, and appointed their representatives on the committee for the nomination of officers as follows:

*Vermont*, J. N. Stiles; *Massachusetts*, John Homans; *Connecticut*, L. N. Beardsley; *New York*, Jos. McNaugeton; *New*

*Jersey*, John Blain; *Delaware*, H. F. Askew; *Ohio*, W. S. Battles; *Indiana*, James F. Hibbard; *Pennsylvania*, Wm. Mayberry; *Michigan*, H. O. Hitchcock; *Kansas*, D. W. Stormont; *Virginia*, John C. Hupp; *Iowa*, J. H. W. Baker; *Wisconsin*, H. Van Dusen; *Illinois*, H. Noble; *Tennessee*, W. K. Boring; *Maryland*, Dr. Cox; *The Army*, Josiah Simmons.

The retiring Acting President, Dr. W. Jewell, then delivered his valedictory address, in which he alluded to the present unhappy condition of the country; paid an eloquent tribute to the memory of the deceased President, Dr. Eli Ives; adverted to the history of the Association, and urged especially upon their attention the subject of Hygiene.

The address was exceedingly well received, and the thanks of the convention voted the author.

Drs. Walter Hay, Thomas Bevan, John McAllister, John Bartlett, M. O. Heydock, Neil P. Peterson, R. C. Hamill, H. N. Hurlbut, and H. Webster Jones, of Chicago; E. C. Lardner, of Vermont; S. W. Bicknell, Beloit; E. W. Jenks, of Sturges; Henry Durham, La Salle; Silas Earle, Onarga; and W. W. Sedgewick, of Sandwich, were elected permanent members, and the following as members by invitation: A. L. Merriam, Sandwich, Ill.; L. D. Glazebrook, St. Pierre, Ind.

The President then appointed Drs. Pearson, Beardsley, and Cutler, to draft resolutions expressive of the sense of the association respecting the death of the late President.

The convention then adjourned till 3 o'clock.

Afternoon.—The convention reässembled at 3 o'clock, and the Committee on Nominations was called upon to make their report. They recommended the following for officers:

*President*—Dr. Alden March, of New York.

*Vice Presidents*—Drs. James Cooper, of Delaware; David Prince, of Illinois; C. C. Cox, of Maryland; and E. S. Carr, of Wisconsin.

*Treasurer*—Dr. Caspar Wistar, of Philadelphia.

REPORT OF THE TREASURER.

The report of the Treasurer was read by Dr. Haskell, of Delaware, the Treasurer being unable to attend. He reported that, owing to the unsettled state of the country and the advanced price of printing, it would be necessary to print only such papers as were of great value, and to condense those as much as possible, or the treasury could not bear the cost. The proceeds of volumes sold were $1,982.25. Balance on hand this year, $504.21. The report was adopted.

The Committee on Publication reported the result of their labors during the year and the number of volumes now in their possession. The report was accepted.

Samuel R. Percy, M. D., Prof. Mat. Med, &c., in the N. Y. Medical College, was announced as the author of an Essay to which the usual prize was to be awarded. Subject—Veratrum Viride and its alkaloid Veratria. Ordered to be printed in the transactions.

Dr. E. K. Squibb, of N. Y., was allowed until the next meeting to report on the "Practical Workings of the U. S. Law relating to the inspection of drugs and medicines."

Other committees were also continued.

Dr. A. K. Gardner, of N. Y., presented a paper on the "Use and Abuse of Pessaries."

The Committee on the Hunter Memorial reported $357 contributed in one-dollar subscriptions.

The Committee of Arrangements proposed the following gentlemen to be elected as permanent members:

Drs. Daniel B. Brengle, Manchester; Van Courtland Secord, Galena; J. B. Samuel, Carrolton; David Dodge, Chicago; James S. King, Lemont; D. F. Crouse, Mount Carroll; all of Illinois. The nominations were confirmed.

### THE USE OF MERCURY BY THE ARMY SURGEONS.

Dr. Lawson called attention to the recent order of the Surgeon General prohibiting the use of mercurials and tartarized antimony by the army Surgical corps. He moved that the society express its disapprobation of the order. The subject was referred to a committee, with instructions to inquire into the facts and report, the committee to consist of one member from each state.

MEDICAL PROVISION FOR RAILROAD ACCIDENTS.

Remarks were then made by Dr. Arnold, of New York, on the necessity of making medical provision for railroad accidents. He distributed printed copies of papers read by him before the State Medical Society and the Academy of Medicine, both of New York.

ARMY SURGEONS.

Dr. Cox called attention to the want of a recognition of army Surgeons, and urged that relative rank should be accorded to them. At present it was not possible for a Surgeon to rise above the rank of major. He therefore offered the following resolution:

*Resolved*, That a committee of five be appointed by the chair to draft a memorial to Congress asking the enactment of a law by which Surgeons in the service of the United States army may be accorded relative rank in the same.

*Resolved*, That each medical gentleman present be urgently invited to use every proper influence with the members of Congress from his respective district, to urge the passage of a law, favorable to this object, at the ensuing session of Congress.

JUNE 3.—The following gentlemen were admitted as members of the Association, by invitation:

Dr. J. H. Foster, Libertyville, Ill.; W. G. Millar, Rockford, Ill.; J. A. Brown, Kankakee City, Ill.

The following permanent members of the Association were elected:

Tiffin Sinks, Leavenworth, Kansas; W. C. Hall, Fayetteville, O.; Hiram Wanzer, Chicago, Ill.; H. K. Dean, Maunkport, Ind.; H. C. Robbins, Newark, Ill.; E. J. Duffield, Woodstock, Ill.; W. Jaynes, Yankton, Dakota Ter.; C. M. Clark, Galva, Ill.

New York City was selected as the next place of meeting.

A committee was appointed to report on the army ambulance system.

A resolution on Compulsory Vaccination was referred to a committee.

The Committee on Voluntary Communications presented the abstract of a paper by Prof. E. Andrews, on "Diatheses—their Surgical Relations"—referred to Com. of Publication.

The meeting by sections was abolished.

Afternoon.—Dr. D. J. McGowan addressed the Convention on medical matters in China and Japan.

The following physicians were elected permanent members:

I. B. Buchtell, South Bend, Ind.; C. Truesdale, Rock Island, Ill.; W. R. Fox, Wilmington, Ill.; L. F. Warner, and M. Parker, of Chicago. Drs. L. T. Hewins, Oak Alla, Wis., and C. J. Taggart, Beloit, were elected members by invitation.

Dr. C. C. Cox, from the Committee on Medical Education, read an able scientific paper on the subject, reviewing the past history of the profession in this respect, and the absence of proper attention to the subject. Many valuable suggestions as to needed improvements, were also made. After the rendering of this report the Committee submitted the following resolutions, which after discussion were adopted:

*Resolved*, That a thorough preliminary education in English, Latin, mathematics and physics, constitutes an essential pre-requisite to the admission of a student of medicine into the office of a medical preceptor, or as a matriculent of a respectable medical college.

*Resolved*, That the advancement of medical education demands a more extended and symmetrical course of instruction in the colleges, and a more thorough and impartial examination for the degree of doctor of medicine than at present prevail.

*Resolved*, That Medical Jurisprudence and Hygeine are highly important branches of Medical Science, deserving the careful consideration of all medical teachers and schools.

*Resolved*, That societies for medical improvement—State, district and county, are important auxiliaries to the advancement and promotion of science, and are therefore highly recommended by this body, as valuable levers in the cause of medical education.

The Committee on the Surgeon General's Order (Dr. Lawson's motion) brought in a majority and minority report, which were respectively discussed in animated language.

June 4.—The following gentlemen were admitted members by invitation:

Isaac Snyder, Jackson, Mich.; *R. B. Treat*, Janesville, Wis.

The following were admitted as permanent members:

Granville S. Thomas, Joliet, Ill.; J. S. Pashley, Osceola, Ill.

Dr. Cox, of the army, announced the sudden departure of Dr. Wilson Jewell, of Pennsylvania, caused by receiving intelligence of the unexpected death of a son, and offered a resolution of condolence which was adopted.

Regular business being in order, the reports of Committees were taken up.

Dr. Gilbert, of the army, in behalf of the Committee on the Extinction of the Aboriginal Races, reported progress, and on motion, the Committee was continued another year.

The resolutions with reference to the Surgeon General's Order were taken up. Dr. Cox, of the army, explained the apparent necessity of the order. The discussion became general, but eventually the report of the majority of the committee, with their appended resolutions, understood to be from the pen of Prof. Lawson, the chairman, were adopted. The resolutions are as follows:

*Resolved*, That from evidence within our possession, we can but entertain the conviction that the Surgeon General of the U. S. Army has been led into expressions, in Order No. 6, which will convey errors respecting the abuse of calomel in the army, and we feel called upon to protect, so far as in our power, the reputation of the intelligent and self-sacrificing medical officers, from the implied imputation of such general mal-practice.

*Resolved*, That while regarding spanæmic medicines, particularly calomel and tartar emetic, when freely administered to soldiers in the field, the camp or hospital, where unfavorable hygienic conditions so commonly cause depressed and asthenic conditions of the system, as being very often productive of injuries; yet that these articles, when judiciously employed, are useful, is a proposition according with the general opinion of the profession; and as abuse of an article is no just argument against its proper use, it is, in the judgment of this body, to be regretted that the object of correcting these abuses was not sought to be effected by an order of caution on the subject, and by dismissing from the service those disregarding such caution, and not by the extraordinary and, as we think, unjustifiable course of attempting to prevent, entirely, the use of articles, though liable to abuse, as are all other powerful agents, yet which are well established in professional confidence as capable of useful application.

On motion, it was resolved that a copy of the above resolutions be forwarded to the President of the United States, the Surgeon General United States Army, and the Secretary of War.

The Nominating Committee reported back the following officers of the Association for the present year:

*Secretaries*—Drs. H. A. Johnson, Ill.; Guido Furman, N. Y.

*Committee of Arrangements*—Drs. James Andrews, N. Blakeman, T. M. Markoe, T. C. Finnell, Austin Flint, Jr., E. S. F. Arnold, J. H. Griscom.

*Committee on Prize Essays*—Drs. D. F. Condie, Pa.; E. Wallace, do.; Wilson Jewell, do; E. R. Peaslee, N. Y.; Alfred Stille, Pa.

*Committee on Medical Education*—Drs. J. C. Dalton, N. Y.; M. L. Linton, Mo.; John Frissell, Va.; Howard Townsend, N. Y.; W. H. Byford, Ill.

*Committee on Medical Literature*—Drs. L. M. Lawson, Ohio; E. L. McGugin, Iowa; William Mayberry, H. Noble, Ill.; John Homans, Mass.

*Committee on Publication*—Drs. F. G. Smith, Chairman, Pa.; Caspar Wistar, do.; Ed. Hartshorne, do; H. S. Askew, Del.; S. G. Hubbard, Conn.; H. A. Johnson, Ill.; Guido Furman, N. Y.

*Committee on Insanity*—Drs. Ralph Hills, Ohio; C. H. Nichols, D. C.; D. P. Bissell, N. Y.; S. W. Butler, Pa.; John S. Butler, Conn.

Dr. H. G. Davis commenced reading a paper on "The American Method of Treating Joint Diseases and Deformities," which was referred to the Committee of Publication, and its further reading suspended.

Dr. Hamburger read a paper upon the use of the laryngoscope, exhibiting the instruments, and another upon a case of disappearance of the iris behind the lens. Referred to Committee of Publication.

The paper of Dr. Griscom, on a case of diarrhœa adiposa, (read on Thursday afternoon,) was, on motion of Dr. Furman, referred to the Committee of Publication.

Dr. A. Fisher read a paper on the use of the sulphites of lime and soda in the treatment of hospital gangrene, phlebitis, erysipelas, and other zymotic diseases. On motion, the paper was referred to a committee of three, of which the author is chairman, to continue his investigation, and report next year.

Dr. Cox, of the Army, offered two resolutions—one of thanks to the citizens of Chicago, for their kindness and hospitality shown to the members of the Association during its sessions here, and another of thanks to the retiring Secretary, Dr. Hubbard, for his able and faithful services.

The amendments to the Constitution of the Association, proposed at the last meeting, were called up and discussed, and so far carried as to fix the time of the next meeting on the first Tuesday of next June.

A complimentary resolution, thanking the President and Secretary for their services, was adopted.

The following gentlemen, on motion of the Committee of Arrangements were elected permanent members of the Association:

L. H. Cary, Toledo, Iowa; Horatio Hitchcock, Chicago; L. F. Warne, do.; L. P. Cheney, do.; C. W. Shumway, do.

Afternoon.—Several committees were allowed further time to report. Dr. N. S. Davis offered an amendment to the Constitution, providing for the election of a Permanent Secretary.

The following resolution was offered by Dr. Arnold and passed:

Whereas, The railroad is fast becoming the great medium of land travel in all parts of the world; and whereas, in spite of all regulations and care, serious accidents are continually occurring, attended with loss of life, such being greatly augmented by the total want of any local medical provision to meet such, as well as by the absence of any appliances whatever, calculated to strengthen the hands of the surgeon; therefore, be it

*Resolved*, That such medical provision should be made by railroads; that by the diminution of suffering, as well as by the saving of life, while economy would acrue to the railroad companies, the interests of humanity would be greatly served.

A lengthy memorial was received and read from the special committee appointed to address Congress in relation to the rank and the pay of army surgeons. On motion, the report was accepted and adopted.

On motion, the Secretary was instructed to have the memorial printed, and to send copies of the same to public officers at Washington.

After some further proceedings of an informal nature, the Convention adjourned.

